# The Mechanism of Intralipid®-Mediated Cardioprotection Complex IV Inhibition by the Active Metabolite, Palmitoylcarnitine, Generates Reactive Oxygen Species and Activates Reperfusion Injury Salvage Kinases

**DOI:** 10.1371/journal.pone.0087205

**Published:** 2014-01-30

**Authors:** Phing-How Lou, Eliana Lucchinetti, Liyan Zhang, Andreas Affolter, Marcus C. Schaub, Manoj Gandhi, Martin Hersberger, Blair E. Warren, Hélène Lemieux, Hany F. Sobhi, Alexander S. Clanachan, Michael Zaugg

**Affiliations:** 1 Postdoctoral Fellow, Cardiovascular Research Centre, University of Alberta, Edmonton, AB, Canada; 2 Research Associate, Department of Anesthesiology & Pain Medicine, University of Alberta, Edmonton, AB, Canada; 3 Research Associate, Department of Clinical Chemistry, University Children’s Hospital Zurich, Zurich, Switzerland; 4 Professor, Institute of Pharmacology and Toxicology, University of Zurich, Zurich, Switzerland; 5 Research Associate, Department of Pharmacology, University of Alberta, Edmonton, AB, Canada; 6 Head of the Department of Clinical Chemistry, University Children’s Hospital Zurich, Zurich, Switzerland; 7 Undergraduate student, Campus Saint-Jean, University of Alberta, Edmonton, AB, Canada; 8 Assistant Professor, Campus Saint-Jean, University of Alberta, Edmonton, AB, Canada; 9 Assistant Professor and Director of Coppin Center for Organic Synthesis, Coppin State University, Baltimore, Maryland, United States of America; 10 Professor, Department of Pharmacology, University of Alberta, Edmonton, AB, Canada; 11 Professor, Department of Anesthesiology & Pain Medicine, University of Alberta, Edmonton, AB, Canada; Jr; University of Pecs Medical School, Hungary

## Abstract

**Background:**

Intralipid® administration at reperfusion elicits protection against myocardial ischemia-reperfusion injury. However, the underlying mechanisms are not fully understood.

**Methods:**

Sprague-Dawley rat hearts were exposed to 15 min of ischemia and 30 min of reperfusion in the absence or presence of Intralipid® 1% administered at the onset of reperfusion. In separate experiments, the reactive oxygen species (ROS) scavenger N-(2-mercaptopropionyl)-glycine was added either alone or with Intralipid®. Left ventricular work and activation of Akt, STAT3, and ERK1/2 were used to evaluate cardioprotection. ROS production was assessed by measuring the loss of aconitase activity and the release of hydrogen peroxide using Amplex Red. Electron transport chain complex activities and proton leak were measured by high-resolution respirometry in permeabilized cardiac fibers. Titration experiments using the fatty acid intermediates of Intralipid® palmitoyl-, oleoyl- and linoleoylcarnitine served to determine concentration-dependent inhibition of complex IV activity and mitochondrial ROS release.

**Results:**

Intralipid® enhanced postischemic recovery and activated Akt and Erk1/2, effects that were abolished by the ROS scavenger N-(2-mercaptopropionyl)glycine. Palmitoylcarnitine and linoleoylcarnitine, but not oleoylcarnitine concentration-dependently inhibited complex IV. Only palmitoylcarnitine reached high tissue concentrations during early reperfusion and generated significant ROS by complex IV inhibition. Palmitoylcarnitine (1 µM), administered at reperfusion, also fully mimicked Intralipid®-mediated protection in an N-(2-mercaptopropionyl)-glycine -dependent manner.

**Conclusions:**

Our data describe a new mechanism of postconditioning cardioprotection by the clinically available fat emulsion, Intralipid®. Protection is elicited by the fatty acid intermediate palmitoylcarnitine, and involves inhibition of complex IV, an increase in ROS production and activation of the RISK pathway.

## Introduction

Intralipid® is the brand name of the first safe fat emulsion for human use, which was invented by the Swedish medical doctor Arvid Wretlind and approved for clinical use in 1962. Beside phospholipids (1.2%) and glycerol (2.2%), it consists of a mixture of neutral triglycerides. The major fatty acid constituents are linoleic acid (C18∶2, ∼60%), oleic acid (C18∶1, ∼30%) and palmitic acid (C16∶0, ∼10–15%). In the clinical setting, Intralipid® is mainly used for parenteral nutrition and serves as solvent of many lipophilic drugs, which would otherwise be insoluble in aqueous solutions and could not be injected intravenously [1]. As rescue therapy, Intralipid® was also found to accelerate detoxification of overdosed lipophilic drugs such as local anesthetics, drugs targeting the central nervous system and various Ca^2+^ channel blockers by acting as a “lipid sink” [2]. More recently, Rahman and colleagues [3] reported marked protection of the heart against ischemia-reperfusion injury with a 70% reduction in infarct size when Intralipid® was added at high doses (1% in the isolated heart or 5 mL/kg body weight *in vivo*) at the onset of reperfusion. That study and a follow-up study [4] from the same group observed activation of protection signaling (Akt, ERK1/2) and phosphorylation (and inhibition) of GSK3β with subsequent increased Ca^2+^ retention capacity of mitochondria, suggesting inhibition of the permeability transition pore. However, the fundamental mechanisms causing this marked protection of the heart remain unclear to date.

Increasing fatty acid oxidation under ischemia/reperfusion conditions is usually regarded as detrimental, while increasing glucose oxidation and concomitantly reducing fatty acid oxidation (Randle cycle) is considered beneficial [5], [6], [7]. In contrast to this “iconic principle” in cardioprotection by metabolic interventions, we have recently reported that infarct-remodeled hearts with limited oxidative capacity accelerate fatty acid oxidation for ATP production and not glucose oxidation after conditioning against ischemia/reperfusion injury [8]. Other studies also support the importance of fat oxidation under conditions of ischemia/reperfusion [9], [10], [11] and raise the possibility that by increasing energy supply, administration of Intralipid® at the onset of reperfusion could help secure energy production and enhance postischemic recovery. Consistent with this concept is the notion that increased fatty acid oxidation plays a beneficial role in the recovery of Intralipid®-rescued hearts exposed to lethal doses of local anesthetics [12]. While such an energy boost from fatty acid oxidation may foster postischemic recovery, mechanisms previously reported for many types of postconditioning should also be considered in Intralipid®-mediated cardioprotection. Therefore, we hypothesized that the formation of reactive oxygen species (ROS) due to inhibition of the electron transport chain would activate reperfusion injury salvage kinases (RISK) or signal transducer and activator of transcription 3 (STAT3) [13], [14]. This hypothesis has not been tested so far and the precise mechanisms of Intralipid®-induced protection remain elusive. Hence we set out to investigate the activities of the respiratory complexes as possible targets of Intralipid®, and further explored the role of ROS. Based on the concept of a “gradual wake-up” [13], we specifically hypothesized that Intralipid^®^ would inhibit electron flux and thus generate ROS at the onset of reperfusion.

Our study now demonstrates that cardioprotection by Intralipid®, observed as improved postischemic LV mechanical function, is caused by increased ROS production during early reperfusion and is accompanied by an accumulation of acylcarnitines. The palmitic acid constituent of Intralipid® initiates postconditioning by inhibition of the respiratory complex IV, generation of ROS and activation of RISK. Our data provide a novel metabolic foundation of Intralipid®-mediated cardioprotection.

## Materials and Methods

All materials were from Sigma-Aldrich (St. Louis, MO, USA) unless otherwise stated. cis-9-octadecenoyl-L-carnitine (oleoylcarnitine, C18∶1, cisΔ-9) and cis,cis-9,12-octadecadienoyl-L-carnitine (linoleoylcarnitine C18∶2, cis,cisΔ-9,12) were not commercially available and synthesized separately as detailed below.

### Synthesis of *cis*-9-octadecenoyl-L-carnitine (Oleoylcarnitine, C18∶1, *cis-9*)

*Cis*-9-octadecenoyl chloride was first prepared by reacting 1.05 mM *cis*-9-octadecenoic acid (from Sigma Aldrich- St. Louis, MO) with 2.1 mM thionyl chloride in 30 ml hexane, and the reaction mixture was heated to 60–80°C in a hot oil bath for 2.5 hours, while stirring, under nitrogen gas flow. The *cis*-9-octadecenoyl chloride was distilled to remove all low boiling point components, hexane and the excess thionyl chloride. Second, the perchlorate salt of the L-carnitine was prepared by reacting 0.20 mM L-carnitine in 25 ml acetonitrile with 0.24 mM silver perchlorate and the reaction mixture was stirred for one hour at room temperature, and the supernatant was collected by decanting. The *cis*-9-octadecenoyl chloride from the first step was added to the supernatant from the second step and the reaction mixture was stirred at 40°C (hot water bath) for 22 hours. The reaction mixture was centrifuge at 2,000 rpm for 10 min at 4°C, and the supernatant was collected and the pH was adjusted to 4 by the addition of sodium bicarbonate. The supernatant was centrifuged at 2,000 rpm for 10 min at 4°C, to remove the sodium bicarbonate. The supernatant was again collected by decanting, and the acetonitrile was evaporated using nitrogen gas till dryness. The *cis*-9-octadecenoylcarnitine product was collected, washed four times using ether 25 ml each time, and the ether was evaporated and the product was collected, with a 88.26% yield of *cis*-9-octadecenoylcarnitine as oily solid component.

### Synthesis of *cis, cis*-9, 12-octadecadienoyl-L-carnitine (Linoleoylcarnitine, C18∶2, *cis, cis*-9, 12)

Linoleoyl chloride (*cis, cis*-9, 12-octadecadienoyl chloride) was first prepared by reacting 1.8 mM *cis, cis*-9, 12-octadecadienoic acid in 25 ml hexane with 5.0 mM thionyl chloride and the reaction mixture was heated to 60–75°C in a hot oil bath for 2.5 hours, while stirring, under nitrogen gas flow. The *cis, cis*-9,12-octadecadienoyl chloride was distilled to remove all low boiling point components, hexane and the excess thionyl chloride. Second, the perchlorate salt of the L-carnitine was prepared by reacting 2.5 mM L-carnitine in 35 ml acetonitrile with 3.0 mM silver perchlorate and the reaction mixture was stirred for one hour at room temperature, and the supernatant was collected by decanting. The *cis, cis*-9,12-octadecadienoyl chloride from step I was added to the supernatant from the second step and the reaction mixture was stirred at room temperature for 60 hours. The reaction mixture was centrifuge at 2,000 rpm for 10 min at 4°C, and the supernatant was collected and the pH was adjusted to 4.5 by the addition of sodium bicarbonate. The supernatant was centrifuged at 2,000 rpm for 10 min at 4°C, to remove the sodium bicarbonate. The supernatant was again collected by decanting, and the acetonitrile was evaporated using nitrogen gas till dryness. The product of *cis,cis*-9,12-octadecadienoylcarnitine (linoleoylcarnitine) was collected, washed five times using ether 20 ml each time, and the ether was evaporated and the product was collected, with a 82.17% yield of *cis,cis*-9,12 octadecadienoylcarnitine as oily component.

### Animal Ethics Statement

The investigation conforms to the Guide for the Care and Use of Laboratory Animals published by the US National Institutes of Health (NIH Publication, 8th Edition, 2011) and was approved by the University of Alberta Animal Policy and Welfare Committee.

### Working Rat Heart Perfusion Protocols

Male Sprague-Dawley rats (350–400 g) were anesthetized with pentobarbital (150 mg/kg, intraperitoneally). Each heart was rapidly removed and perfused initially in a non-working Langendorff mode with Krebs-Henseleit solution for 15 min. The working mode perfusion was subsequently established (11.5 mmHg preload, 80 mmHg afterload, 5 Hz) with a recirculating perfusate of 100 mL (37°C, pH 7.4) gassed with 95% O_2_/5% CO_2_ mixture that consisted of a modified Krebs-Henseleit solution containing (mmol/L): KCl (4.7), NaCl (118), KH_2_PO_4_ (1.2), MgSO_4_ (1.2), CaCl_2_ (2.5), NaHCO_3_ (25), glucose (11), palmitate (1.2, pre-bound to 3% bovine serum albumin) and insulin 100 mU/L [8]. All hearts were subjected to 15 min of 37°C zero-flow ischemia and 30 min of reperfusion. Longer periods of global ischemia would have resulted in no functional recovery at reperfusion, which would have markedly biased all metabolic measurements and measurements of metabolic flux rates. Cardiac output (mL/min) and aortic flow (mL/min) were measured using ultrasonic flow probes (Transonic T206, Transonic Systems Inc., Ithaca, NY) placed in the left atrial inflow and the aortic outflow lines. Left ventricular work (mL/min·mmHg) was calculated as LVW = cardiac output • (aortic systolic pressure − preload). Measurements of mechanical function were averaged for the pre- and postischemic periods. Hearts were assigned to the following four groups 1) IR = untreated without Intralipid® (Baxter, Mississauga, ON) 2) IR/IL = Intralipid® 1% administered immediately at the onset of reperfusion 3) IR/IL+MPG = Intralipid® 1% plus 10 µM N-(2-mercaptopropionyl)-glycine concomitantly administered at the onset of reperfusion, 4) IR/MPG = 10 µM N-(2-mercaptopropionyl)-glycine alone. 1 µM palmitoylcarnitine (IR/C16∶0c), linoleoylcarnitine (IR/C18∶2c) or oleoylcarnitine (IR/C18∶1c) were added to the perfusate at the onset of reperfusion in separate experiments to mimic Intralipid® protection. In a separate set of experiments with Intralipid®, reperfusion time was reduced to 3 minutes. Finally, additional groups of hearts were perfused aerobically (time-matched, 30 min) in the absence or presence of Intralipid® 1% to explore the direct effects of Intralipid® on mitochondrial respiration and oxidative metabolism in absence of ischemic injury. Cardiac fibers were collected from the apex of the left ventricle at the end of the perfusions. All hearts were immediately frozen in liquid nitrogen with Wollenberger clamps and stored at −80°C for subsequent molecular analyses. Additional hearts were flushed of blood and used to collect fibers to measure complex IV inhibition, mitochondrial H_2_O_2_ release and uncoupling in response to acylcarnitines *in vitro*.

### High-resolution Respirometry in Permeabilized Cardiac Fibers

Respiration measurements were performed in saponin-permeabilized fibers [8] prepared from freshly excised left ventricular apex of either perfused or unperfused hearts using the Oroboros Oxygraph 2 K system (Oroboros, Innsbruck, Austria). Measurements were conducted in an assay solution containing 110 mM sucrose, 60 mM K-lactobionate, 20 mM taurine, 0.5 mM EGTA, 3 mM MgCl_2_·6 H_2_O, 10 mM KH_2_PO_4_, 20 mM HEPES, and 1 g/l BSA (pH 7.1 at 30°C). Characterization of the mitochondrial respiratory complexes was obtained using the following substrates in the presence of 5 mM ADP or absence of ADP (leak respiration): pyruvate (5 mM)/malate (2 mM), succinate (10 mM), ascorbate (2 mM)/tetramethylphenylenediamine dihydrochloride (TMPD; 0.5 mM), and palmitoylcarnitine (20 µM)/malate (2 mM). Substrates used together with the inhibition of complex I by rotenone (0.5 µM), complex III by antimycin A (2.5 µM), and complex IV by azide (100 mM) provided complex-specific flux measurements. Proton leak in permeabilized cardiac fibers was measured as detailed in the supplementary file Protocol S1.

### Citrate Synthase Activity

The activity of the mitochondrial matrix marker enzyme citrate synthase (CS) was measured at 412 nm by monitoring the formation of thionitrobenzoate, as previously described [15].

### Mitochondrial Complex III Activity

The assay was performed using MitoTox OXPHOS complex III activity kit (MitoSciences, Eugene, OR). Mitochondrial protein was isolated from tissue homogenates. After centrifugation at 10,000 g for 15 min 4°C, the resulting pellet was resuspended for protein concentration determination. 2–3 µg of mitochondrial protein was used in a 96-well plate for complex III activity measurement by monitoring the conversion of oxidized cytochrome c to its reduced form, as indicated by the linear increase in the absorbance at 550 nm.

### Amplex Red Assay for the Determination of Mitochondrial Hydrogen Peroxide (H_2_O_2_) Release in Permeabilized Cardiac Fibers

To assess mitochondrial H_2_O_2_ emission capacity from collected cardiac fibers, we determined H_2_O_2_ production under non-respiring conditions and under active oxidative phosphorylation (5 mM ADP) using a combination of pyruvate (5 mM)/malate (2 mM) and succinate (10 mM) as substrates to ensure full operation of the citric acid cycle, as well as to provide both NADH and FADH_2_ to feed electrons through complexes I and II of the mitochondrial electron transport chain, respectively. H_2_O_2_ production was measured fluoroscopically using Amplex Red (Invitrogen, Carlsbad, CA) [16]. Horseradish peroxidase (HRP; 2 U/ml) catalyzed the reaction between Amplex Red (20 µM) and H_2_O_2_ in the presence of exogenously added superoxide dismutase (10 U/ml), forming the fluorophore resorufin, which was monitored at excitation/emission wavelengths of 540/590 nm (Synergy H4 hybrid multi-mode microplate reader, BioTek Instruments, Inc. Winooski, VT USA). H_2_O_2_ standard curves were generated for each independent experiment, to calculate the cumulative mitochondrial H_2_O_2_ production from the resorufin signal. H_2_O_2_ production at each time point was then determined by calculating the rate of change in H_2_O_2_ concentration over 20 min. Background rates of fluorescence change in the absence of added substrates were subtracted for each experiment. At the end of each experiment, cardiac fibers were collected and homogenized for citrate synthase (CS) activity assay. H_2_O_2_ production rate was expressed as pmol/min/IU CS activity.

### Aconitase Assay for the Determination of ROS Production in Mitochondrial Matrix of Perfused Hearts

Aconitase activity was measured according to the protocols of Gardner et al. [17] from mitochondria isolated from perfused hearts. Mitochondria were isolated in the presence of 5 mM sodium citrate to protect aconitase from oxidation. Mitochondrial preparations were diluted to 0.1 mg/ml in 50 mM Tris-HCl, pH 7.4 containing 0.05% Triton X-100. Aconitase activity was assayed as the rate of NADP^+^ reduction (molar extinction coefficient for NADPH at 340 nm is 6,220 M^−1^cm^−1^) at 37°C in a reaction mixture containing 50 mM Tris-Cl (pH 7.4), 5 mM sodium citrate, 0.6 mM MnCl_2_, 0.2 mM NADP^+^, 1 U/ml isocitrate dehydrogenase, and 0.1 mg/ml mitochondrial protein. One unit of aconitase converted 1.0 nmol of citrate to isocitrate per minute at 37°C.

### Immunoblotting for Akt, STAT3, and ERK1/2 and Enzyme-linked Immuno-absorbent Assay for Akt Activity

Frozen heart tissue was homogenized in ice-cold buffer containing 50 mM Tris (pH 8.0), 150 mM NaCl, 1% Nonidet P-40, supplemented with protease and phosphatase inhibitor cocktail mix (Sigma-Aldrich). The homogenate was centrifuged at 1000 g for 10 min at 4°C. The resulting supernatant was collected for protein concentration determination (Bradford assay) and used for immunoblotting in sodium dodecyl sulfate polyacrylamide gel electrophoresis (SDS-PAGE). The primary antibodies for Akt, STAT3 and ERK1/2 were rabbit anti-Akt, rabbit anti-STAT3 and rabbit anti-ERK1/2 (all 1∶500) and rabbit anti-pAkt, rabbit anti-pSTAT3 and rabbit anti-pERK1/2 (all 1∶500) (Cell Signaling Technology, Danvers, MA). Tubulin (mouse monoclonal, Sigma-Aldrich, T6074) was used as loading control. Immunoreactivity was visualized by horseradish peroxidase-conjugated antibodies using a peroxidase-based chemiluminescence detection kit (ECL) (PerkinElmer, Woodbridge, Ontario, Canada). The intensity of the bands was quantified using ImageJ** software. The total Akt activity was determined from cytosolic fractions based on solid phase enzyme-linked immuno-absorbent assay from Enzo (Enzo Life Sciences, Farmingdale, NY). Frozen heart tissue was homogenized in buffer containing 10 mM Hepes (pH 7.4), 1.5 mM MgCl_2_, 10 mM KCl, 0.3 M sucrose, 0.05% Nonidet P-40, and 5 mM 1,4-dithiothreitol supplemented with protease and phosphatase inhibitor cocktail mix. After centrifugation at 10,000 g for 15 min 4°C, the resulting supernatant was subjected to protein concentration determination (Bradford assay). The color reaction was measured in a microplate reader at 450 nm.

### Mass Spectrometry for Acylcarnitine Profiling

Tissue levels of acylcarnitine species were measured using electrospray ionization tandem mass spectrometry [7]. Acylcarnitines were extracted from heart tissue with methanol and quantified using eight isotopically labeled internal standards (Cambridge Isotopes Laboratories, Andover, MA). Precursor ions of m/z 85 in the mass range of m/z 150 to 450 were acquired on a PE SCIEX API 365 LC-ESI-MS/MS instrument (AppliedBiosystems, Foster City, CA).

### Oxidative Metabolism: Contribution of Intralipid^®^ to Fatty Acid Substrate Metabolism in Perfused Hearts

In separate experiments, competition for β-oxidation between albumin-bound radiolabeled palmitate and fatty acid constituents released from Intralipid® was determined in aerobically perfused hearts [18] to estimate Intralipid®’s contribution to substrate metabolism and to further evaluate a possible effect on glucose-fatty acid oxidation partitioning (Randle cycle) [19]. Glucose and fatty acid oxidation were determined by perfusing the hearts with [U-^14^C]glucose and [9,10-^3^H]palmitate, respectively. Total myocardial ^14^CO_2_ production and ^3^H_2_O production were determined every 10 min. Rates expressed as µmol/g dry wt/min were calculated for each time interval and were averaged for 30 min of perfusion.

### Statistical Analysis

Values are given as mean (SEM) or median (25^th^, 75^th^ percentile) depending on the underlying data distribution for the indicated number of independent observations (n). The significance of differences in hemodynamic and metabolic variables among groups was determined by Student *t-*test (two groups) or by analysis-of-variance (ANOVA) followed by the Student-Newman-Keuls method for posthoc analysis or by non-parametric methods (Mann-Whitney rank sum test or Kruskal-Wallis test) depending on the underlying data distribution. Differences are considered significant if p<0.05. The inhibitory effect of palmitoylcarnitine and linoleoylcarnitine on respiratory chain complex IV activity was measured by nonlinear curve fitting of a 4-parameter logistic function to the experimental data as follows:
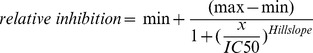



IC_50_, is the half maximal inhibitory concentration, Hillslope is the slope of the concentration-response curve, max and min are extreme values (no inhibition and maximum inhibition, respectively). SigmaPlot Version 12 (Systat Software, Inc., San Jose, CA 95110 USA) was used for all analyses.

## Results

### Cardioprotection by Intralipid® Administered at the Onset of Reperfusion is ROS-dependent and is Mimicked by Palmitoylcarnitine

**Figure 1:**
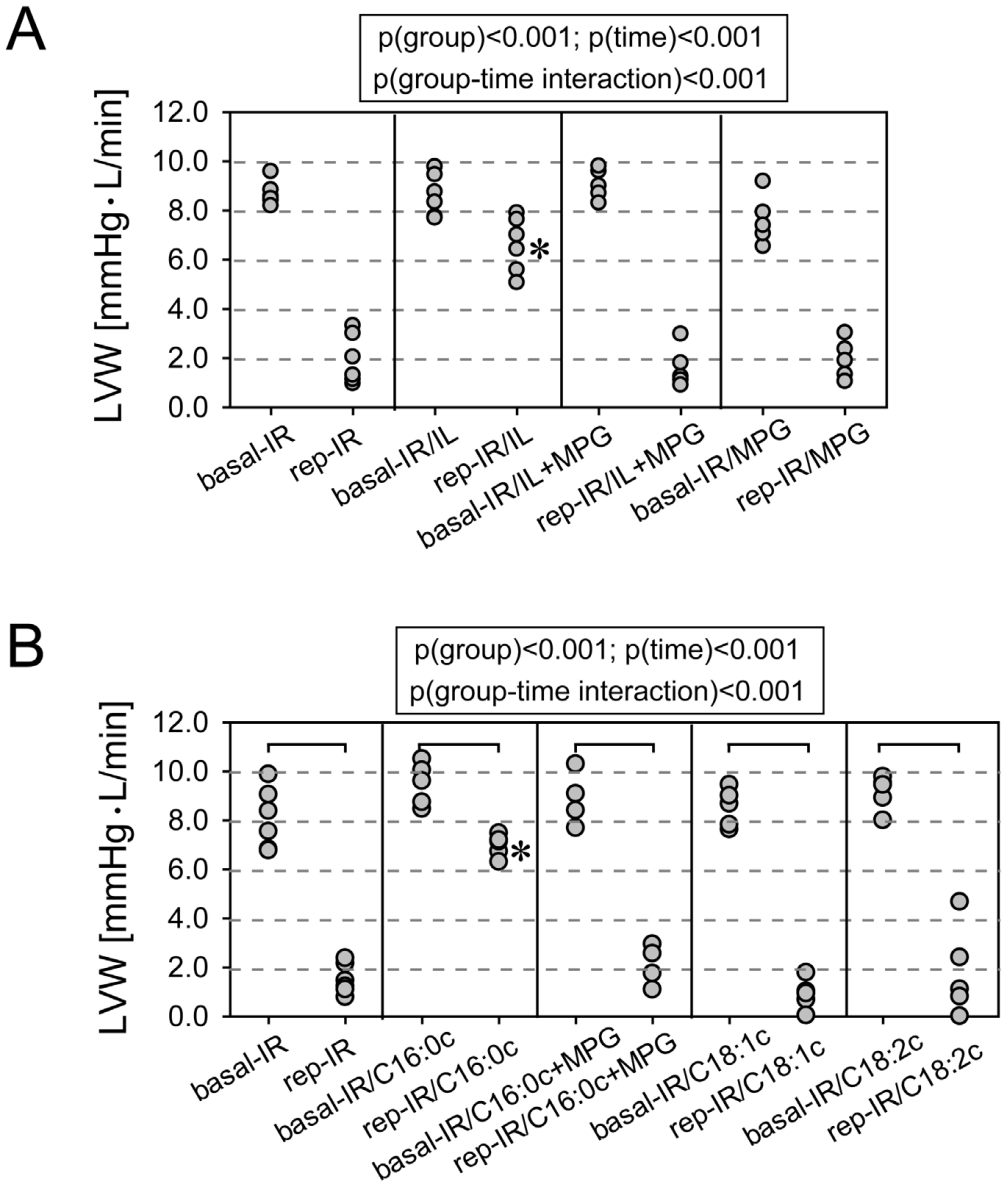
Functional recovery in Intralipid®-treated (Panel A) and acylcarnitine-treated hearts (Panel B) subjected to 15 min of ischemia and 30 min of reperfusion. LVW, left ventricular work. Basal, average LVW before ischemia. Rep, average LVW during postischemic reperfusion. IR, untreated hearts exposed to 15(IR). IR/IL, hearts exposed to IR and 1% Intralipid® at the onset of reperfusion. IR/C16∶0c, IR/C18∶1c, and IR/C18∶2c, hearts exposed to IR and 1 µM palmitoyl-, oleoyl-, or linoleoylcarnitine at the onset of reperfusion. MPG, N-(2-mercaptopropionyl) glycine (10 µM) added at the onset of reperfusion. *, significantly different from all other groups. N = 6–7 hearts in each group.

Hearts treated with Intralipid® (1% administered at the onset of reperfusion) showed marked recovery (79%±2% of baseline) compared with untreated hearts (24%±1%) (Figure 1A). Administration of pamitoylcarnitine (1 µM) at the onset of reperfusion fully mimicked Intralipid® protection (74%±2% of baseline; Figure 1A). However, administration of linoleoylcarnitine (1 µM), oleoylcarnitine (1 µM) or glycerol (0.11 g per 100 mL as in a 1% Intralipid® emulsion) did not result in any protection (glycerol: 33%±9%). The ROS scavenger MPG abolished both Intralipid®- and pamitoylcarnitine-mediated cardioprotection (Figure 1).

### ROS Released from the Mitochondrial Electron Transport Chain Trigger Activation of Akt and ERK1/2 during Early Reperfusion in Intralipid®-treated Hearts

**Figure 2:**
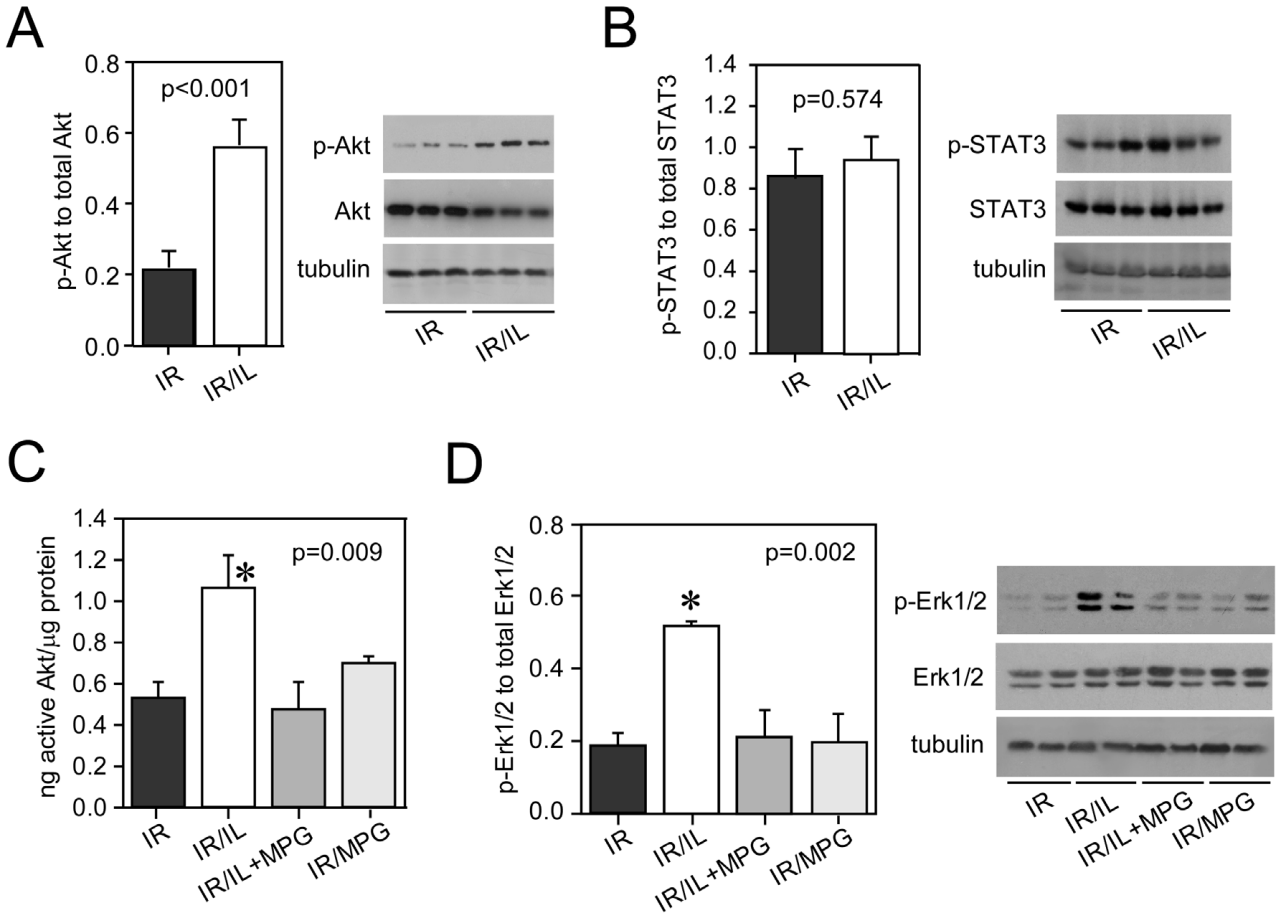
Early activation of Akt and ERK1/2 by ROS in Intralipid®-treated hearts. Panel A: p-Akt to total Akt immunoblots from tissue samples collected 3 min after reperfusion. Panel B: p-STAT3 to total STAT3 immunoblots from same tissue samples. Panel C: Akt activity measurements. Panel D: p-ERK1/2 to total ERK immunoblots from same tissues. IR, untreated hearts exposed to 15 min of ischemia and 3 min of reperfusion (IR). IR/IL, hearts exposed to IR and 1% Intralipid® at the onset of reperfusion. MPG, N-(2-mercaptopropionyl) glycine (10 µM) added at the onset of reperfusion. Data are mean (SEM). N = 6 hearts in each group.

**Figure 3:**
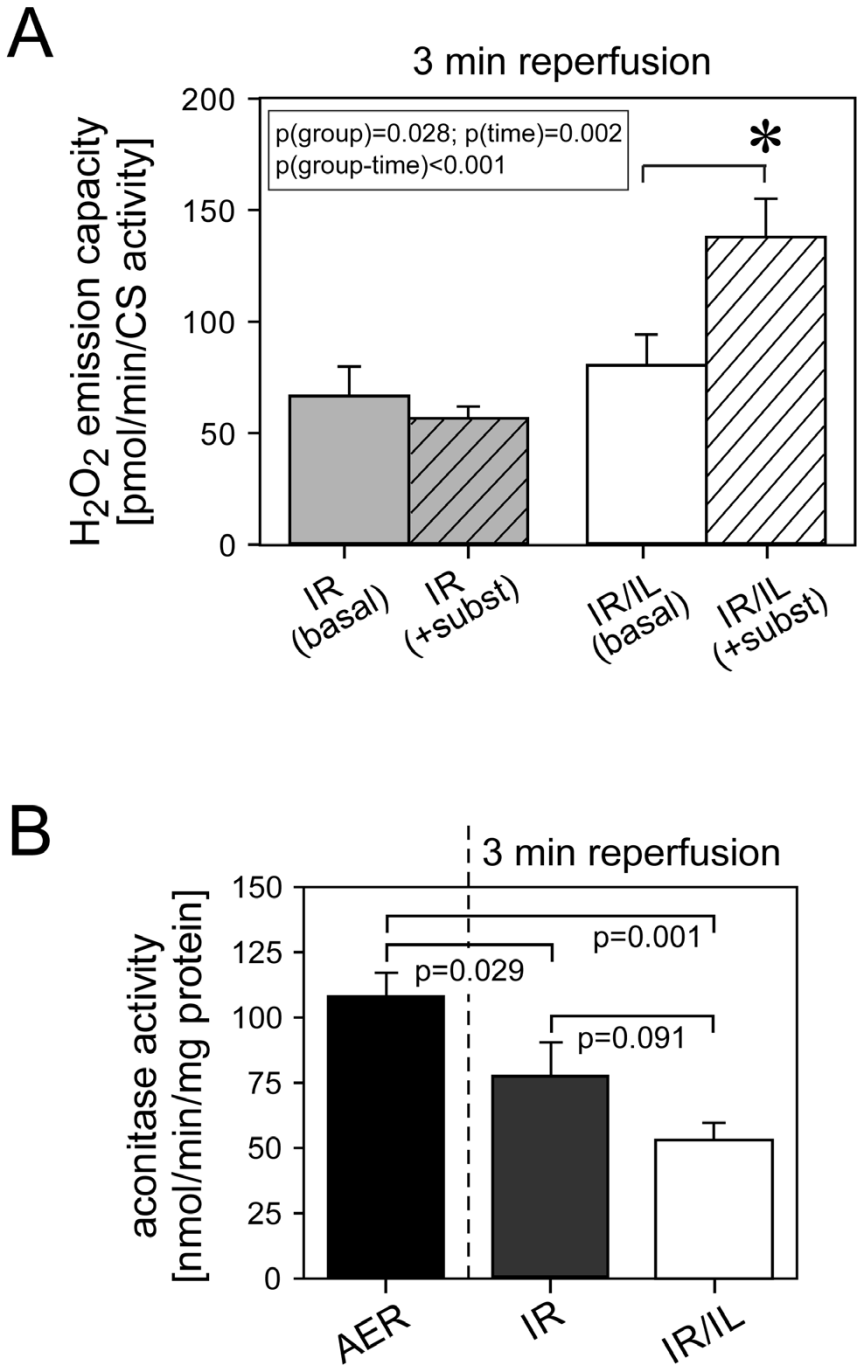
Intralipid®-induced formation of ROS in cardiac fibers collected 3 min after reperfusion. Panel A: Hydrogen peroxide (H_2_O_2_) emission capacity from mitochondria as determined by Amplex Red assay. *significantly different from IR. Panel B: Loss of aconitase activity in Intralipid®-treated hearts (P_ANOVA_ = 0.002). IR, untreated hearts exposed to 15 min of ischemia and 3 min of reperfusion (IR). IR/IL, hearts exposed to IR and 1% Intralipid® at the onset of reperfusion. Basal, without substrates. +subst, with added substrates. AER, hearts with time-matched aerobic perfusion. Data are mean (SEM). N = 6 hearts in each group.

Although previous work identified RISK, i.e. Akt and ERK1/2, as mediators of Intralipid®-induced cardioprotection [3], the mechanism of RISK activation remains unclear. We hence tested whether ROS would be required to activate RISK or STAT3. Rat hearts were either untreated or were exposed to Intralipid® 1%, Intralipid® plus MPG or MPG alone at the time of reperfusion. Intralipid® activated Akt (Figure 2A) but not STAT3 (Figure 2B) at early reperfusion (3 min). ROS scavenging with MPG prevented activation of Akt (Figure 2C and 2D) and abolished protection (Figure 1). Administration of MPG alone did not affect Akt activation. Likewise, Intralipid® activated ERK1/2, which was abolished by MPG (Figure 2D). Similar to Intralipid®, palmitoylcarnitine-induced protection was strongly ROS-dependent, i.e. inhibited by the ROS scavenger MPG (Figure 1). We then determined H_2_O_2_ emission capacity from energized and non-energized mitochondria of cardiac fibers collected after 3 min of reperfusion in untreated and Intralipid®-treated hearts using Amplex Red in the presence of exogenously added Cu/Zn superoxide dismutase. This assay detects superoxide released from the entire electron transport chain into the intermembrane space and the mitochondrial matrix in response to metabolic stimulation. Our experiments showed a marked ROS burst from mitochondria in hearts treated with Intralipid® at the time of early reperfusion, concomitant with RISK activation (Figure 3A). Loss of aconitase activity, a specific and sensitive marker of ROS production within the mitochondrial matrix, revealed only slightly lower aconitase activity consistent with some matrix-released ROS formation at 3 min of reperfusion in Intralipid®-treated hearts (Figure 3B).

### Palmitoylcarnitine Inhibits Mitochondrial Electron Transport Chain at Complex IV in a Concentration-dependent Manner and Induces ROS Production

**Figure 4:**
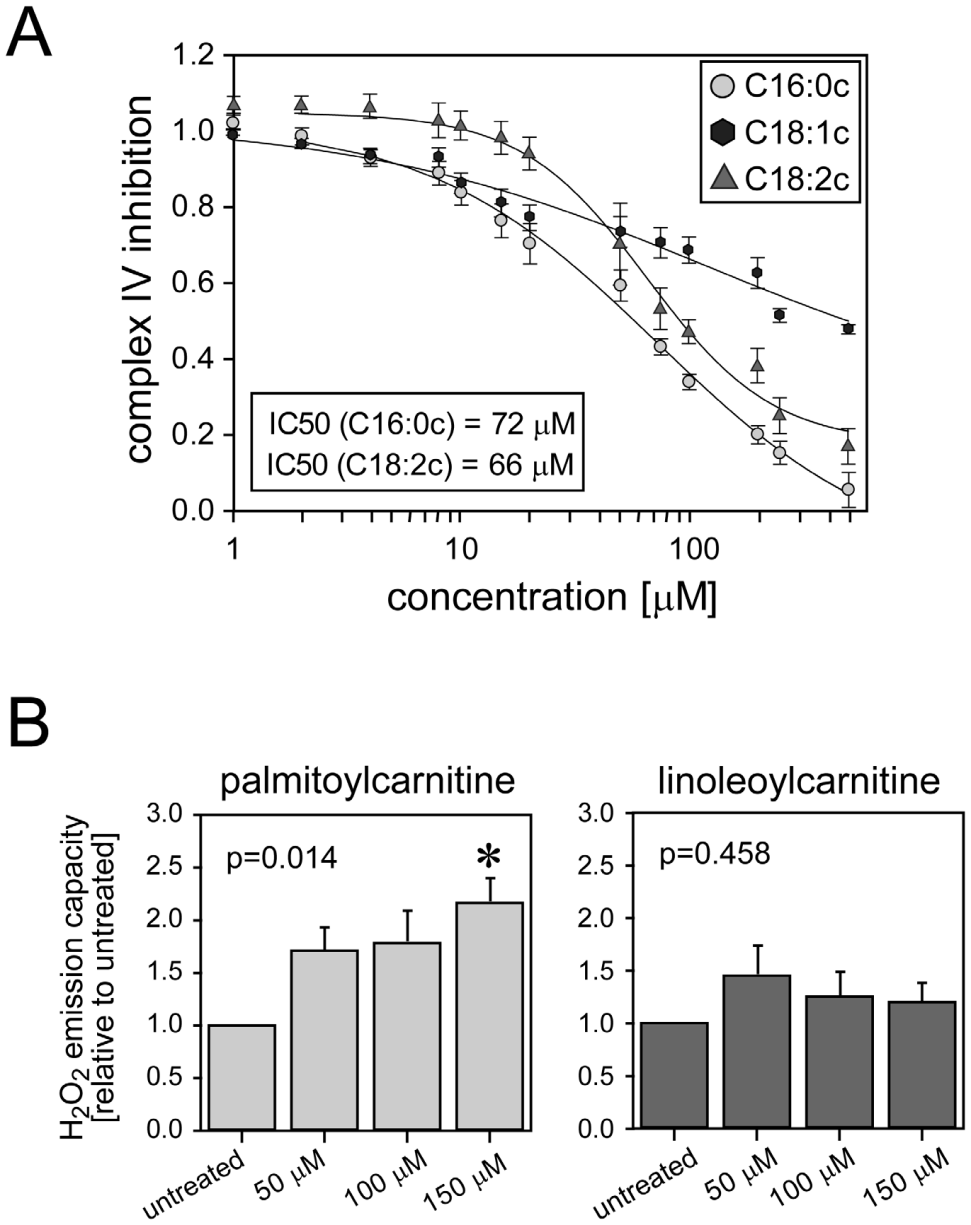
Complex IV inhibition by metabolites of Intralipid® fatty acid constituents. Panel A: Concentration-dependent inhibition of complex IV by palmitoyl(C16∶0c)-, oleoyl(C18∶1c)-, and linoleoylcarnitine (C18∶2c) in permeabilized cardiac fibers. Complex IV inhibition is given as relative decrease in oxygen consumption. Panel B and C: Concentration-dependent hydrogen peroxide (H_2_O_2_) emission capacity determined by Amplex Red assay in permeabilized cardiac fibers exposed to increasing concentrations of palmitoyl- and linoleoylcarnitine. *, significantly different from untreated. Data are mean (SEM). N = 6 hearts in each group/concentration.

Inhibition of the respiratory chain activity promotes ROS production from mitochondria and is known to be a common basic mechanism of cardioprotection [13]. To identify the site of mitochondrial ROS production in Intralipid®-treated hearts, we measured mitochondrial enzymes activities (either polarographically or spectrophotometrically) in permeabilized cardiac fibers from rat hearts aerobically perfused in the presence and absence of Intralipid^®^ 1%. Intralipid® did not decrease complex I, II and III activities, but markedly decreased complex IV activity (Table 1, Table S1). The fact that complex IV inhibition did not affect overall oxygen consumption when using complex I and complex II substrates is well known and represents an inherent feature of the mitochondrial respiratory chain to maintain a constant electron flow despite variations in complex IV activity [20]. To test which of the fatty acid constituents of Intralipid® would elicit inhibition of complex IV activity, we used acylcarnitines of palmitic (C16∶0c), oleic (C18∶1c) and linoleic acids (C18∶2c), the main constituents of Intralipid^®^ and intermediates of fatty acids, which directly access mitochondria. These acylcarnitines were titrated to permeabilized cardiac fibers energized with complex IV substrates to establish the concentration-dependent inhibition of complex IV. Palmitoyl- and linoleoylcarnitine induced concentration-dependent inhibition of complex IV activity (Figure 4A). The IC_50_ values for palmitoyl- and linoleoylcarnitine were quite similar (Table 2). Conversely, oleoylcarnitine inhibited complex IV activity only modestly at very high concentrations (500 µM) (Figure 4A). Complex IV inhibition by palmitoylcarnitine (150 µM) increased H_2_O_2_ release from energized cardiac fibers (Figure 4B). Linoleoylcarnitine (150 µM) also slightly increased H_2_O_2_ release, but this did not reach statistical significance (Figure 4B).

**Table 1:** Assessment of mitochondrial respiratory chain function.

Substrate/electron donor	Site(s) of electron input	Method		Results	
			AER	AER/IL	p-value
pyruvate/malate	Complex I (NADH:ubiquinone oxidoreductase)	HRR	8.3 (0.6)	7.0 (0.4)	0.074
pyruvate/malate/succinate	Complex I+II	HRR	10.7 (0.8)	12.2 (0.8)	0.243
succinate	Complex II (succinate:ubiquinone oxidoreductase)	HRR	6.1 (0.7)	7.8 (0.5)	0.080
decylubiquinol	Complex III (ubiquinol:ferricytochrome c oxidoreductase)	SP	8.5 (0.7)	8.1 (0.3)	0.652
ascorbate/TMPD	Complex IV (cytochrome c oxidase)	HRR	22.2 (1.6)	16.3 (1.5	**0.024**
palmitoylcarnitine/malate	Complex I+Electron transfer flavoprotein-ubiquinone oxidoreductase	HRR	2.9 (0.3)	2.6 (0.2)	0.434

Rat hearts were aerobically perfused with/without Intralipid (1%). Complex I, complex II, and complex IV function was measured polarographically by monitoring oxygen consumption in presence of specific substrates and ADP in saponin-skinned cardiac fibers using high-resolution respirometry (HRR). Complex III enzymatic activity was assayed spectrophotometrically (SP). The measured oxygen consumption (normalized to citrate synthase activity) is expressed as nmol O_2_*s^−1^/CS. Complex III activity is expressed as mOD*min^−1^/µg mitochondrial protein. Data are presented as mean (SEM). N = 6–10 in all groups.

Abbreviations:

AER, aerobically perfused hearts without treatment; AER/IL, aerobically perfused hearts exposed to Intralipid (1%); TMPD, tetramethyl-p-phenylene diamine, an artificial electron carrier which is reduced by ascorbate producing electrons that are transferred to cytochrome c; OD, optical density.

**Table 2:** Fitting parameters for the acylcarnitine inhibitor curves.

compound	IC_50_ [µM]	95% CI [µM]	Hillslope	95% CI
Palmitoylcarnitine (C16∶0c)	72.3	(37.7;108)	−0.86	(−1.07; −0.65)
Linoleoylcarnitine (C18∶2c)	65.6	(49.9;81.4)	−1.58	(−1.99; −1.16)
Oleoylcarnitine (C18∶1c)	NA	NA	NA	NA

CI, confidence interval; IC_50_, the inhibitor concentration that reduces the response by 50%. NA, not applicable.

### Intralipid® and Palmitoylcarnitine Similarly Induce “Mild Uncoupling”

**Figure 5:**
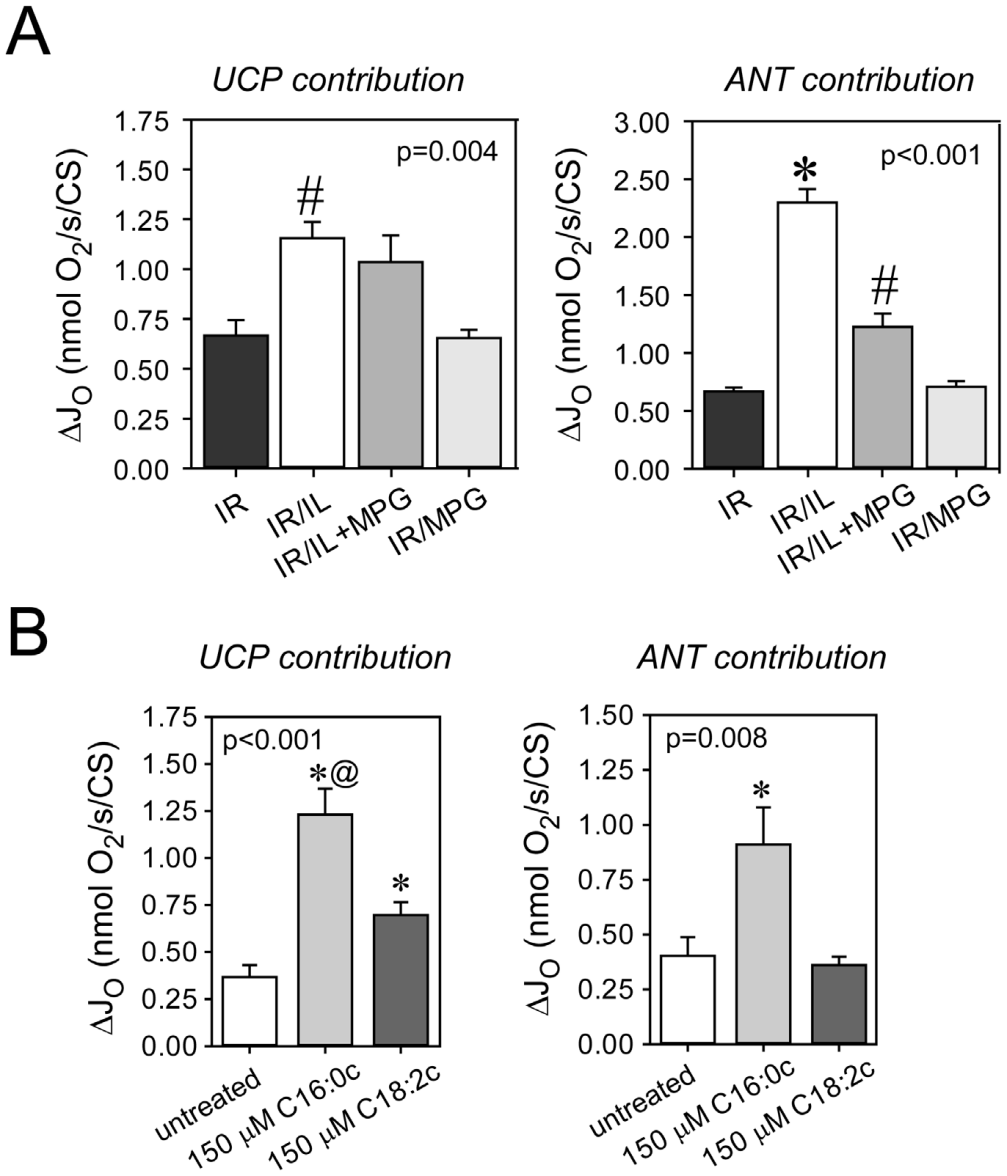
Uncoupling by Intralipid® and acylcarnitines. Panel A: Uncoupling by uncoupling proteins (UCP) and adenine nucleotide translocase (ANT) were measured in cardiac fibers collected at the end of 30 min reperfusion. N-(2-mercaptopropionyl)-glycine (MPG) inhibits ANT- but not UCP-mediated uncoupling. #, significantly different from IR and IR/MPG. *, significantly different from all other groups. Panel B: Cardiac fibers respiring on succinate (leak respiration) were exposed to 150 µM palmitoyl-(C16∶0c) and linoleoylcarnitine (C18∶2c) and UCP- and ANT-mediated uncoupling were determined. IR, untreated hearts exposed to 15 min of ischemia and 30 min of reperfusion (IR). IR+IL, hearts exposed to IR and 1% Intralipid® at the onset of reperfusion. IR/IL+MPG, hearts exposed to IR and 1% Intralipid® +10 µM N-(2-mercaptopropionyl) glycine (MPG) at the onset of reperfusion. *, significantly different from all other groups. @, significantly different from C18∶2c. Data are mean (SEM). N = 6 hearts in each group.

Two mitochondrial carriers – uncoupling proteins (UCP) and the adenine nucleotide translocase (ANT) – display enhanced proton conductance when they are activated by ROS or fatty acids [21], [22], [23]. We thus tested whether Intralipid®-treated hearts would also exhibit increased uncoupling. Cardiac fibers were collected at the end of 30 min reperfusion and UCP- and ANT-mediated proton leak were measured using the specific blockers guanosine diphosphate and carboxyatractyloside, respectively. UCP- and ANT-mediated uncoupling was markedly increased in Intralipid®-treated hearts (Figure 5A). The ROS scavenger MPG diminished ANT- but not UCP-mediated leak, suggesting a possible mechanistic role of leak respiration in the recovery from ischemia-reperfusion injury. Experiments with cardiac fibers directly exposed to palmitoyl- and linoleoylcarnitine confirmed increased uncoupling via UCP by both acylcarnitines and via ANT by palmitoylcarnitine only (Figure 5B).

### Accumulation of Acylcarnitines in Intralipid®-treated Hearts without Contribution to Substrate Metabolism

**Figure 6:**
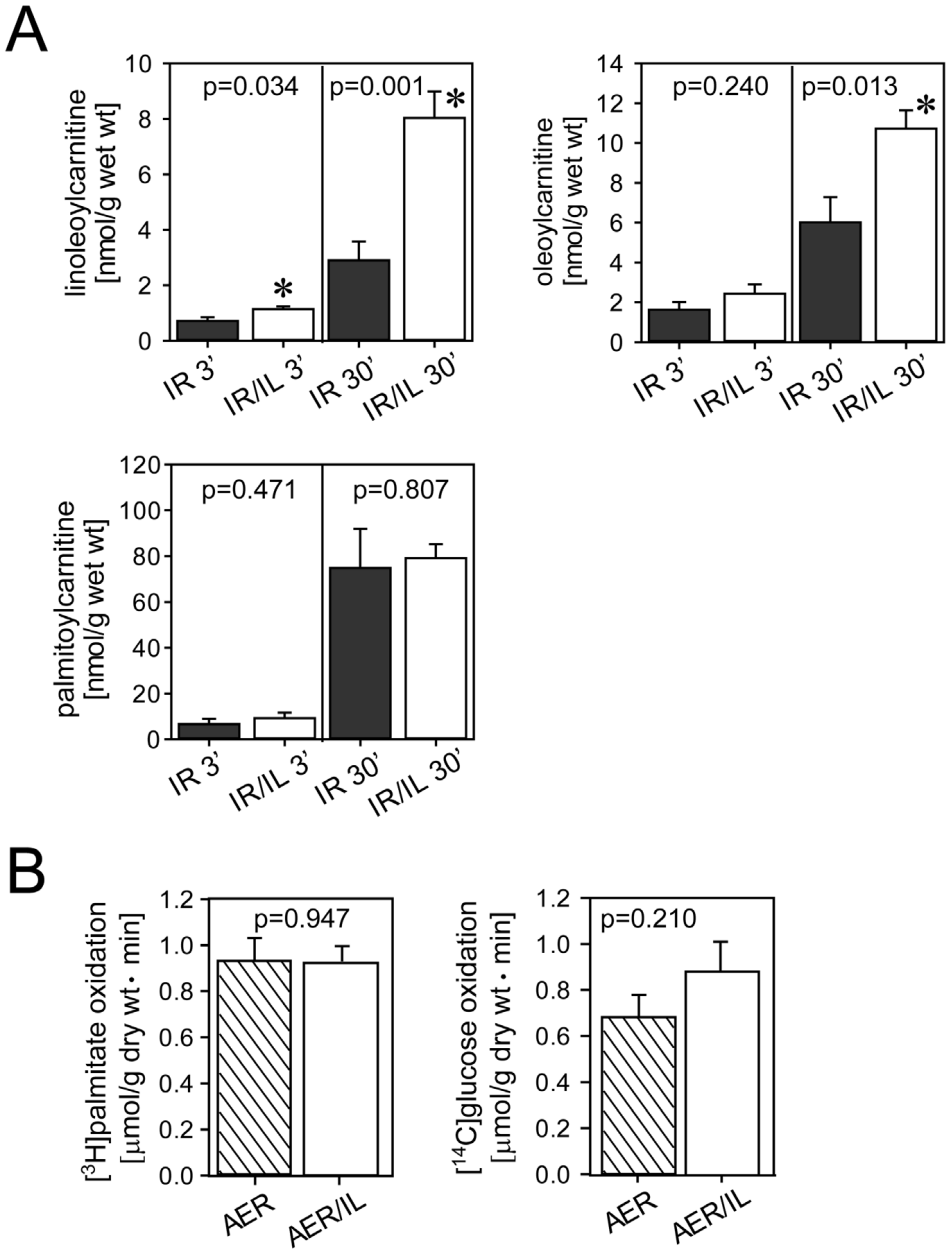
Accumulation of acylcarnitines and effects on oxidative metabolism in Intralipid®-treated hearts. Panel A: Accumulation of long-chain acylcarnitines in hearts reperfused for 3 min and 30 min. Panel B: Lack of competition for β-oxidation between exogenous radiolabeled palmitate and fatty acid constituents released from Intralipid® and no alteration in glucose oxidation (Randle cycle). AER, time-matched aerobically perfused hearts. AER/IL, time-matched aerobically perfused hearts treated with 1% Intralipid® for 30 min. IR 3′ or 30′, untreated hearts exposed to 15 min of ischemia and 3 or 30 min of reperfusion, respectively. IR/IL 3′ or 30′, hearts exposed to 15 min of ischemia and 3 or 30 min of reperfusion and 1% Intralipid® at the onset of reperfusion. *, significantly different from the corresponding untreated group. Data are mean (SEM). N = 6 hearts in each group.

Hearts subjected to ischemia-reperfusion and exposed to Intralipid® 1% accumulated linoleoyl- and oleoylcarnitine starting at early reperfusion (Figure 6A), suggesting mitochondrial uptake of Intralipid®-derived fatty acid constituents. Additional experiments showed that there was no reduced utilization of the radiolabeled albumin-bound palmitate (1.2 mM) in the presence of 1% Intralipid® during aerobic perfusion of hearts at a constant workload [24], implying minimal competition for β-oxidation between albumin-bound and Intralipid®-derived fatty acids and thus no relevant contribution of Intralipid® to substrate metabolism (Figure 6B). Also, there was no decrease in glucose oxidation in hearts exposed to 30 min of 1% Intralipid® perfusion in the presence of 1.2 mM palmitate in the perfusate, i.e. no change in glucose-fatty acid oxidation partitioning (Randle cycle) (Figure 6B).

## Discussion

**Figure 7:**
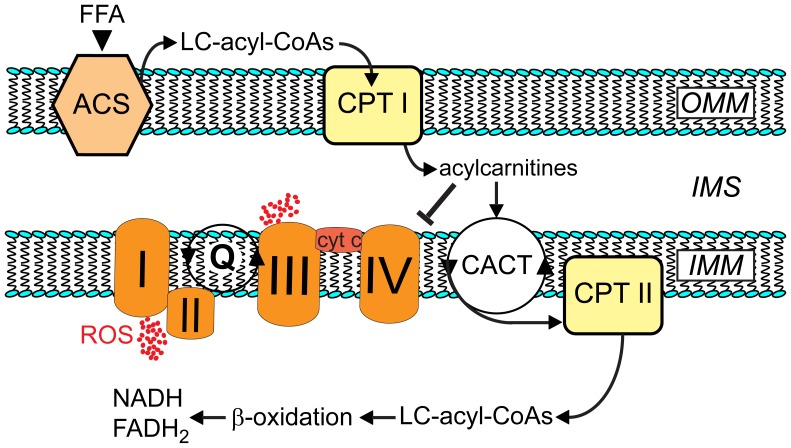
Proposed mechanism of Intralipid®-induced cardioprotection. Metabolites of fatty acid constituents released from Intralipid® accumulate in the intermembrane space (IMS) in early reperfusion when β-oxidation is still dysfunctional. An initial peak of acyl-CoAs in the mitochondrial matrix may also inhibit carnitine/acylcarnitine translocase (CACT) and carnitine palmitoyl transferase II (CPT II) via product feedback inhibition, which would further boost acylcarnitine accumulation in the intermembrane space. Acylcarnitines, namely palmitoylcarnitine, inhibit complex IV of the respiratory chain (causing superoxide release at complex I (toward matrix) and at complex III (toward IMS). ACS, acyl-CoA synthase cyt c, cytochrome *c*. CPT I, carnitine palmitoyl transferase I. FFA, free fatty acids. IMM, inner mitochondrial membrane. LC, long-chain. OMM, outer mitochondrial membrane. Q, Q-junction is the point in the respiratory chain where electron flow from complex I and from complex II converge.

Intralipid®-mediated postconditioning improves recovery of postischemic mechanical function, involves activation of RISK (but not STAT3), and is clearly dependent on ROS formation during early (3 min) reperfusion. Additional experiments revealed a novel metabolic foundation underlying Intralipid®-induced cardioprotection that involves the accumulation of acylcarnitines in mitochondria, which inhibits electron transport at complex IV, generates “protective” ROS and thus activates RISK (see Figure 7). Surprisingly, and in contrast to what one would expect from the fatty acid composition of Intralipid®, the results of our study indicate that the unsaturated fatty acids of the emulsion are not essential to Intralipid®-mediated protection. Rather, cardioprotection is mediated by palmitoylcarnitine arising from the release of palmitate from Intralipid®, a fatty acid that only constitutes ∼10–15% of the emulsion. This conclusion is supported by the following findings. First, administration of palmitoylcarnitine, but not the C18-carnitines, at the time of reperfusion fully mimicked Intralipid®-mediated cardioprotection. Second, fatty acids were released from the emulsion and metabolized to acylcarnitines that accumulated in cardiac tissue. Third, palmitoylcarnitine, but not linoleoylcarnitine or oleoylcarnitine, stimulated ROS production as a consequence of complex IV inhibition. Fourth, cardioprotection by Intralipid® or palmitoylcarnitine were both MPG-dependent indicating an essential role of ROS production in their mechanism of action. Collectively, our data show that inhibition of complex IV during the early (3 min) reperfusion by palmitoylcarnitine generated from Intralipid® elicits a form of pharmacological postconditioning that involves ROS generation and activation of RISK, and is a novel and highly effective mechanism of cardioprotection.

### Is Cardioprotection by Intralipid® Linked to Enhanced Substrate Metabolism?

Intralipid® was first approved clinically for parenteral nutrition and was later found beneficial during cardiac reperfusion [25] where it improved contractility of the stunned canine myocardium when administered 15 min following initiation of reperfusion. Interestingly, oxfenicine, a carnitine palmitoyl transferase-1 inhibitor, abolished this effect suggesting a mechanism involving enhanced energy substrate availability or substrate oxidation. A second study in rabbits [26] confirmed the beneficial effect of Intralipid® and identified two constituents, linoleic acid and phospholipids, as the protective agents. While these observations suggest that provision of additional energy substrates in the form of fatty acids during reperfusion is the underlying mechanism of Intralipid®-mediated cardioprotection, more recent reports from Eghbali’s group indicate activation of Akt/ERK1/2 and inhibition of GSK3β when Intralipid® is administered right at the onset of reperfusion, either in a Langendorff mouse heart model (1% Intralipid®) or in an *in vivo* rat model (20% Intralipid®, 5 mL/kg body weight) [3]. These data are consistent with the concept of postconditioning where RISK are activated during the earliest stages of reperfusion [27] and also consistent with reports showing activation of RISK but not STAT3 in pharmacological as opposed to ischemic postconditioning [28]. Although Eghbali’s group demonstrated a reduction in mitochondrial Ca^2+^ overload and the prevention of the permeability transition pore opening by Intralipid® [3], both representing downstream events of RISK activation, the molecular mechanisms of Intralipid®-mediated protection, specifically the link between Intralipid® as a pharmacologically complex fat emulsion with potential impact on substrate and energy metabolism and the activation of RISK, and ultimately protection itself remained unresolved.

Our experiments now clearly demonstrate that under aerobic and constant workload conditions, the rate of exogenous palmitate oxidation was unaffected by Intralipid® indicating that there was no significant competition for β-oxidation by fatty acids potentially released from Intralipid® by endothelium-bound lipoprotein lipase. Overall fatty acid oxidation was also likely unaffected as there was no change in glucose-fatty acid oxidation partitioning as predicted by the Randle Cycle [19]. However, analysis of tissue acylcarnitine content revealed that triglyceride hydrolysis indeed occurred as tissue contents of linoleoylcarnitine or oleoylcarnitine were significantly higher in Intralipid®-treated hearts. Collectively, these observations question the idea of Intralipid® as a mere enhancement of energy substrate supply.

### Role of ROS Production in Intralipid®-mediated Cardioprotection

The ability of the ROS scavenger, MPG, to prevent Intralipid®-mediated cardioprotection strongly suggests participation of ROS. Indeed, enhanced ROS production due to inhibition of the respiratory chain either by short episodes of ischemia or by pharmacological means is a common mechanism of “conditioning”-mediated cardioprotection [13]. In this concept, ROS generated from electrons leaking from the respiratory chain of mitochondria activate RISK and/or Survivor Activating Factor Enhancing Pathway (SAFE) [27], [28] involved in cardioprotection. We therefore tested whether Intralipid® or its constituents would indeed inhibit specific complexes of the electron transport chain. To avoid interference with the “biochemical threshold effect” [29] inherently functional in respiratory complexes subjected to metabolic stress of ischemia/reperfusion, we determined the effects of Intralipid® on individual respiratory complex activities under aerobic conditions. Intralipid® inhibited exclusively complex IV [30], a target inhibited by other cardioprotective agents such as carbon monoxide or hydrogen sulfide [31], [32]. The release of ROS by Intralipid® was confirmed directly as measured by Amplex Red in the presence of superoxide dismutase. This assay measures superoxide release toward matrix (complex I) as well as intermembrane space (complex III), and was more distinct and robust than when ROS production was determined by the loss of aconitase activity, which exclusively measures superoxide released toward matrix. The topology of superoxide release from multiple sources of the respiratory chain supports the view of a downstream inhibition of the electron flux by Intralipid® at complex IV [33].

Current literature describes both protective and deleterious effects of ROS [34], [35]. A burst of ROS during reperfusion is associated with injury, but a controlled amount of ROS during the preconditioning stimulus or at the onset of reperfusion in a postconditioning protocol triggers protective signaling and reduces ischemia-reperfusion injury [28], [35], [36], [37], [38], [39]. Thus, in the context of ischemia-reperfusion, the effects of ROS are “site-” and “time-sensitive”, i.e. site and time will ultimately determine whether ROS are beneficial or detrimental. Since administration of the postconditioning stimulus has to occur at the onset of reperfusion to be effective and postconditioning protection is ROS-dependent, the concept emerges that postconditioning by Intralipid® and possibly other protective agents promotes production of small amounts of ROS during early reperfusion, but during the course of reperfusion significantly reduces ROS formation, an idea, which is further supported by our observation that Intralipid®-induced ROS production occurred simultaneously with RISK activation. Since administration of MPG to untreated hearts did not result in any cardioprotection, we infer that administration of MPG after successful Intralipid® postconditioning would show the same protection as Intralipid® alone. This has been indeed shown by Penna et al. using the ROS scavenger N-acetylcysteine, but ROS were not measured directly in that study [39].

Interestingly, Intralipid®-induced ROS production was accompanied by enhanced UCP- and ANT-mediated leak respiration [21], [23], [40]. This uncoupling feedback mechanism relieves pressure on the electron transport chain when the membrane potential is high through “leakiness” of the inner mitochondrial membrane and thus prevents continuing production of high rates of potentially noxious ROS [40]. On the other hand, mild uncoupling during reperfusion is beneficial per se and may indeed represent another mechanism by which Intralipid® and palmitoylcarnitine protect the heart against reperfusion injury [41].

### Role of Fatty Acids Released from Intralipid®

Only palmitoylcarnitine mimicked the beneficial actions of Intralipid® while the other two acylcarnitines (i.e., linoleoylcarnitine or oleoylcarnitine), had no cardioprotective action. Importantly, as for Intralipid®-mediated cardioprotection, the beneficial action of palmitoylcarnitine was also abolished by MPG. Indeed, an examination of the direct effects of the acylcarnitines revealed that, although linoleoylcarnitine and palmitoylcarnitine concentration-dependently inhibited complex IV, only palmitoylcarnitine-mediated inhibition of complex IV was accompanied by ROS production. These observations strongly suggest that palmitoylcarnitine is the active cardioprotective metabolite of Intralipid® and is supported by an earlier observation that palmitoylcarnitine enhances postischemic cardiac function [42]. The inability of the unsaturated fatty acid intermediates to increase ROS production as opposed to the saturated palmitoylcarnitine may be explained by their differential effects on respiratory control [43] and antioxidant properties [44], [45]. We have not determined the mechanism by which the low concentration of palmitoylcarnitine (1 µM) is effective despite being administered in the presence of a high concentration of palmitate (1.2 mM). Possibly, palmitoylcarnitine has preferential access to its intramitochondrial target (complex IV) as it by-passes a number of steps required for the access of palmitate; these include release from albumin, transport into the cytoplasm, acylation to palmitoyl CoA prior to conversion into palmitoylcarnitine.

The increase in mitochondrial H_2_O_2_ emission capacity at palmitoylcarnitine concentrations of ∼150 µM suggests accumulation in a microcompartment. In fact, the accumulation of substrate intermediates in microenvironments (microcompartmentation) is a common feature in mitochondria [46], [47], and the existence of multiple substrate pools within mitochondria is thought to be advantageous in metabolic regulation and efficiency [47]. Since β-oxidation is dysfunctional and inhibited by high levels of NADH, FADH_2_ and acetyl-CoA during early reperfusion, accumulation of acyl-CoAs and more so long-chain acylcarnitines is substantial under these conditions [24], [48]. We speculate that administration of additional acylcarnitines during early reperfusion may lead to an initial peak of long-chain acyl-CoAs, which are known to decelerate carnitine/acylcarnitine translocase and carnitine palmitoyl transferase-2 via product feedback inhibition. This would further increase accumulation of acylcarnitines in the intermembrane space in close proximity to complex IV. Unfortunately, relevant changes in metabolite concentrations, in our case of palmitoylcarnitine, occurring exclusively in a microcompartment such as the intermembrane space and/or the inner mitochondrial membrane in a short time window are very challenging to measure and may go undetected. Irrespectively, Tominaga and colleagues [49] reported that palmitoylcarnitine as low as 1 µM administered to permeabilized ventricular rat myocytes hyperpolarized the mitochondrial membrane and induced ROS production, which is in accordance with inhibition of the electron flux in mitochondria. Like our own study, that study used permeabilized myocytes, which provide the unique opportunity to examine mitochondria under more physiological conditions, namely the control of cytosolic acylcarnitine concentrations without changing extramitochondrial Ca^2+^, ATP or other substrates.

The mechanism(s) by which acylcarnitines, and more specifically palmitoylcarnitine, inhibit complex IV remain unclear. Complex IV is a dimeric enzyme of 13 subunits per monomer, which is regulated by availability of substrates, interference with the catalytic cycle, multiple allosteric regulations and covalent posttranslational modifications [30], [50], and organized with other respiratory complexes (namely I and III) in functional supercomplexes (“respirasomes”) [51]. Direct interaction of amphiphatic substances such as palmitoylcarnitine with complex IV was recently reported [52], and as the Hillslope of palmitoylcarnitine-mediated inhibition is not significantly different from −1, the existence of a specific binding site and a bimolecular interaction may be postulated. Clearly, additional molecular studies will be required to unravel the details of palmitoylcarnitine-mediated inhibition of complex IV.

### Limitations

Our model assessed cardioprotection as improved recovery of post-ischemic mechanical function. Although this reflects a model of reversible injury (stunning), it has significant advantages, specifically in view of the metabolism-targeted questions which we wanted to address. Any longer periods of global ischemia would have resulted in no functional recovery at reperfusion, which would have markedly biased all metabolic measurements.

Although only palmitoylcarnitine mimics Intralipid®-mediated cardioprotection in a ROS-dependent manner, it was unexpected that increases in palmitoylcarnitine tissue levels were not detectable in Intralipid®-perfused hearts. Nevertheless, C18∶2-carnitine accumulated in Intralipid®-treated hearts during early reperfusion, and C18∶2-carnitine and C18∶1-carnitine were each increased by 5 nmol/g wet wt at the end of reperfusion. Clearly, such increases in C18∶2-carnitine and C18∶1-carnitine indicate that fatty acids were released from Intralipid®, but as palmitic acid is a low abundance constituent (10–15%) of Intralipid® and as palmitoylcarnitine is likely to accumulate in microcompartments in mitochondria, a difference in total tissue concentrations may not be detectable.

We cannot completely rule out that other constituents of the relatively poorly-defined fat emulsion may also contribute, at least in part, to the observed beneficial effects of Intralipid®. However, in additional experiments we excluded the possibility that glycerol, which is also part of the emulsion (0.11 g per 100 mL in a 1% Intralipid® emulsion), exerts protective effects in the heart.

### Summary

In conclusion, our data describe a new mechanism of postconditioning cardioprotection elicited by the fatty acid intermediate palmitoylcarnitine, and further provide a novel metabolic foundation for cardioprotection by the clinically available fat emulsion, Intralipid®, that involves inhibition of complex IV, an increase in ROS production and activation of the RISK pathway.

## Supporting Information

Table S1 **Complete data on oxygen consumption in saponin-skinned cardiac fibers harvested from rat hearts aerobically perfused with/without Intralipid (1%).**
(PDF)

Protocol S1 **Contributions of uncoupling proteins and of the adenine nucleotide translocase to mitochondrial leak respiration.**
(PDF)
