# Supplementary material for: The Mechanism of Intralipid®-Mediated Cardioprotection Complex IV Inhibition by the Active Metabolite, Palmitoylcarnitine, Generates Reactive Oxygen Species and Activates Reperfusion Injury Salvage Kinases
Source: PLoS One. 2014 Jan 30;9(1):e87205. doi: 10.1371/journal.pone.0087205 (PMC3907505; doi:10.1371/journal.pone.0087205)
Supplement: Table S1 — Complete data on oxygen consumption in saponin-skinned cardiac fibers harvested from rat hearts aerobically perfused with/without Intralipid (1%). (PDF) [file pone.0087205.s001.pdf]

Table S1 Complete data on oxygen consumption in saponin-skinned cardiac fibers harvested from rat hearts aerobically perfused with/without Intralipid (1%).

Oxygen consumption in presence of ADP			
	**AER**	**AER/IL**	**p-value**
pyruvate/malate	8.3 (0.6)	7.0 (0.4)	0.074
succinate	6.1 (0.7)	7.8 (0.5)	0.080
palmitoylcarnitine/malate	2.9 (0.3)	2.6 (0.2)	0.434
Oxygen consumption in absence of ADP (LEAK)			
pyruvate/malate	1.2 (0.3)	1.4 (0.3)	0.152
succinate	2.5 (0.3)	3.6 (0.6)	**0.002**
palmitoylcarnitine/malate	0.5 (0.1)	1.2 (0.3)	**<0.001**
Respiratory Control Ratio			
pyruvate/malate	7.4 (2.5)	6.9 (1.4)	0.599
succinate	2.2 (0.6)	2.2 (0.6)	0.996
palmitoylcarnitine/malate	6.0 (1.2)	2.7 (0.9)	**0.001**

The measured oxygen consumption – normalized to citrate synthase (CS) activity – is expressed as nmol $O_2 * s^{-1}$/CS. The Respiratory Control Ratio is computed as the ratio between the oxygen consumption rates in presence and absence of ADP, respectively. Data are presented as mean (SD) or median (25th percentile;75th percentile). N=6-8 in all groups.

Abbreviations: AER, aerobically perfused hearts without treatment; AER/IL, aerobically perfused hearts exposed to Intralipid (1%).
