# Supplementary material for: The Mechanism of Intralipid®-Mediated Cardioprotection Complex IV Inhibition by the Active Metabolite, Palmitoylcarnitine, Generates Reactive Oxygen Species and Activates Reperfusion Injury Salvage Kinases
Source: PLoS One. 2014 Jan 30;9(1):e87205. doi: 10.1371/journal.pone.0087205 (PMC3907505; doi:10.1371/journal.pone.0087205)

Protocol S1: Contributions of uncoupling proteins and of the adenine nucleotide translocase to mitochondrial leak respiration.

Mitochondrial respiration measurements were performed in saponin-permeabilized fibers prepared from freshly excised left ventricular apex of perfused hearts, using the Oroboros Oxygraph 2K system (Oroboros, Innsbruck, Austria). Measurements were conducted in an assay buffer comprising 110 mM sucrose, 60 mM K-lactobionate, 20 mM taurine, 0.5 mM EGTA, 3 mM $MgCl_2 \cdot 6H_2O$, 10 mM KH_2PO_4, 20 mM HEPES, and 1 g/l BSA (pH 7.1 at 30C). To discriminate the various components of proton leak, permeabilized cardiac fibers were first incubated in the presence of 10 mM succinate, 0.5 μM rotenone, and 2 μg/ml oligomycin to obtain the protons leak rates before sequentially adding 0.5 mM guanosine diphosphate (GDP) and 5 μM carboxyatractyloside (CAT) to selectively inhibit uncoupling proteins (UCPs) and ANT. GDP- and CAT-sensitive proton leak rates are indicative of UCPs and the adenine nucleotide translocase (ANT) proton leak activity, respectively. All respiratory data were normalized to citrate synthase (CS) activity (nmol O/s/CS). The complete protocol is illustrated in the following figure.

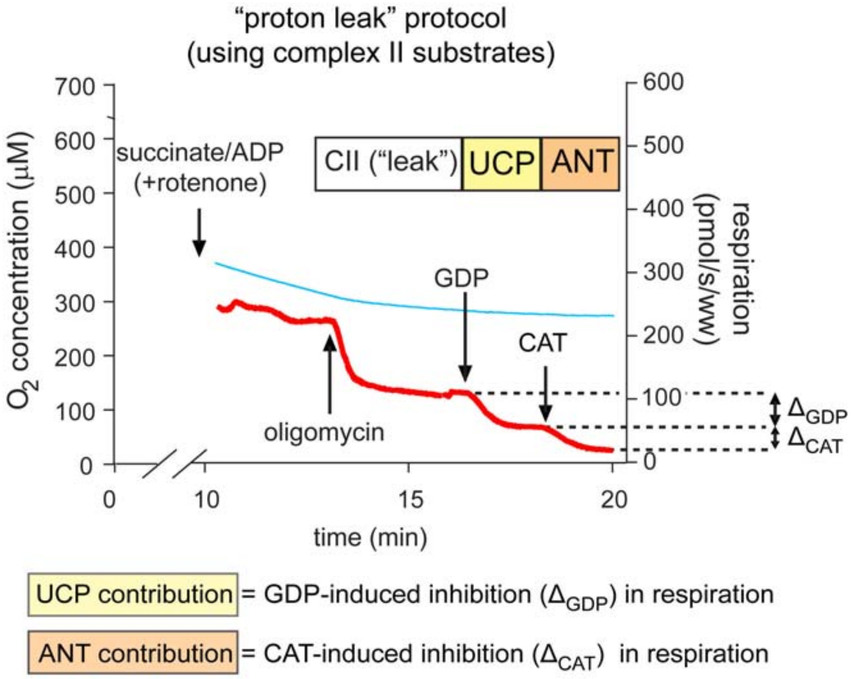

Supplement: Protocol S1 — Contributions of uncoupling proteins and of the adenine nucleotide translocase to mitochondrial leak respiration. (PDF) [file pone.0087205.s002.pdf]
